# Risk of ionizing radiation in pregnancy: just a myth or a real concern?

**DOI:** 10.1093/europace/euac158

**Published:** 2022-09-20

**Authors:** Majdi Saada, Erick Sanchez-Jimenez, Ariel Roguin

**Affiliations:** Cardiology Department, Hillel Yaffe Medical Center, Technion – Israel Institue of Technology, Ha-Shalom St, Hadera 3810101, Israel; Cardiology Department, Hillel Yaffe Medical Center, Technion – Israel Institue of Technology, Ha-Shalom St, Hadera 3810101, Israel; Cardiology Department, Hillel Yaffe Medical Center, Technion – Israel Institue of Technology, Ha-Shalom St, Hadera 3810101, Israel

**Keywords:** Angiography, Cardiology, Pregnancy, Radiation hazards, Women

## Abstract

There are natural concerns regarding the risks posed to the foetus by ionizing radiation exposure during pregnancy. Therefore, many female physicians select to avoid working in an environment associated with ionizing radiation exposure like the catheterization laboratory and even exclude training as electrophysiology, interventional cardiologists, or radiologists. For those already working in this field, pregnancy involves usually a 1-year interruption (pregnancy and maternity leave) to their careers, leading at times to delays in the decision to become pregnant. This review describes the low added risk of malformation/cancer in the offspring, highlight gaps in our understanding, discuss several common wrong beliefs, and recommend how to further decrease radiation dose, especially during pregnancy.

## Introduction

Almost one-third of women of childbearing age, reported radiation exposure as a barrier to choosing interventional cardiology and radiology.^1^ This was the most commonly identified barrier among women <40 years of age.^2^ In the past few decades the number of women in many schools of medicine grew up to 50% or more, and women can be seen today in all fields of medicine. Women constitute 42% of internal medicine trainees, but when it comes to cardiology fellowships, just 23% are women. Women constitute only a small percentage of physicians in the field of fluoroscopically guided interventions (FGI), such as electrophysiology (EP) interventional radiology and interventional cardiology.

It is known that the developing foetus is radiation sensitive, based primarily on mammalian animal studies.^3^ Concerns related to radiation exposure, especially during pregnancy, are a major barrier to recruiting women into these fields.^4^

Especially in a review dealing with radiation risks and pregnancy, it must be emphasized that even in the general population without any exposure to radiation, the success rate of becoming pregnant within a year is only 80–85% and declines with the women’s age. It is estimated that 15% of known pregnancies end in spontaneous abortion. Approximately 2–4% of babies are born with congenital malformations or abnormalities.^3^ Although multifactorial, the rate of learning disability and disorders and especially attention and concentration problems are among the most common behaviour control difficulties exhibited by children and adolescents, and their rate increased significantly in recent decades.

Still, how can someone blame that the success of becoming pregnant, not having a spontaneous abortion, or a congenital defect and later on if there is a diagnosis of a learning difficulty or any behavioural problem in your sibling; was not related to radiation exposure of the mother while working in the catheterization laboratory? The goal of this review is to present the available literature regarding the effects of radiation on childbearing and pregnancy, with a focus on FGI, in an effort to emphasize what is known, highlight gaps in our understanding and discuss several common wrong beliefs.

## Background radiation

We are all exposed to ‘background’ radiation as part of our daily activities. This background radiation comes from the environment ground, soil, and water and from cosmic radiation. A small fraction of background radiation comes from man-made sources, such as radioactive elements emitted from nuclear reactors, radioactive materials used in industry, and even in some consumer products such as tobacco, building materials (cement, granite, and glazed tiles), and antiques (clothes, jewellery, books, and furniture).^5^

The average background radiation dose is 1.0–2.3 milli-sievert (mSv).^1^ The dose varies depending on geography and altitude where a person lives. A report on radiation exposure of the UK population^2^ indicated that the average annual dose to cardiologists was 0.12 mSv, which is well below the occupational annual dose limit of 20 mSv and also well below the annual average background radiation dose.

Of note, the developing foetus in the general population, encounters radiation exposure ubiquitous to the maternal environment. Background radiation during a typical pregnancy is 0.75–1 mSv, though it varies based on geographic location, airline travel, and work environment.^6,7^

## Radiation measurement and reporting

Radiation exposure can be expressed as ‘absorbed dose’ or ‘kerma’ that represents the energy of absorbed radiation by an organ or tissue per unit of mass, typically measured in milli-gray (mGy). In biology, the term ‘equivalent dose’ (ED) is used, which takes into account the radiation weighting factor relevant for the biological effect of the absorbed radiation in a specific organ. It is expressed in mSv. For X-rays, mGy and mSv are numerically equivalent. Radiation exposure is expressed in SI units (see Supplementary material online, *Table S1*).^8,9^

Fluoroscopy systems commonly report the kerma area product (KAP) expressed in mGy× cm^2^, which is defined as the product of air kerma (the sum of the initial kinetic energies of all charged particles released by uncharged ionizing radiation, divided by the mass of the sample) and the area of the radiograph field at the location of the interventional reference point. The KAP describes the total energy incident upon a patient. Dose area product (DAP) values integrate many factors and takes into account the degree of collimation, pulse frequency (and hence, number of frames), and dose per pulse.

The most used factor to convert DAP or KAP values into estimated ED for male adults is 0.20 [ED (mSv) = 0.20 × DAP (mGy·cm^2^)]. For women and children, the conversion factor is higher since they have a higher risk of cancer development by radiation.^10,11^ One study found a corrected factor for pregnant workers of 0.27, 0.23, and 0.17 in the first, second, and third trimesters, respectively.^12^ Radiation exposure estimated as ED (based on DAP values) is a pragmatic approach to roughly compare the relative radiation risks to patients. Yet, the calculated ED remains an estimate with a large intrinsic inaccuracy of ±50%.

In general, the foetal dose of radiation is often described as a tissue dose, although this is not always uniform. Foetal radiation <50 mSv is generally considered negligible based on studies demonstrating that foetal exposure to a dose of <50 mGy does not affect pregnancy outcome compared with control populations.^13–15^

In the USA, the National Council on Radiation Protection and Measurements (NCRP) recommends limiting occupational radiation exposure of the foetus to no more than 5 mSv during the entire pregnancy and 0.5 mSv per month of pregnancy. In the UK and Norway, the levels of allowable radiation exposure during pregnancy effectively preclude women from continuing to practice during gestation.^16^ These regulations presume that despite adequate shielding, radiation exposure to the foetus is ≈50% of the mother's exposure. In most European countries, foetal dose limit is set at 1 mSv/pregnancy, which is the same as the dose limit for the general population.^14,17^

## Risks and effects of radiation

### Risk of infertility

The sensitivity of the reproductive organs to ionizing radiation may raise concern among interventional cardiologists regarding the effects of occupational exposure on the ability to conceive. The dosimeter reading worn under the lead apron at waist level can be used as an indicator of gonadal exposure. It is worth noting that the under-lead dosimeter overestimates gonadal exposure as it does not account for radiation attenuation by the individual's abdominal tissues. Experimental studies in animals have shown that doses of <100 mSv will have no effect on the embryo/foetus neither in the preimplantation stage, nor in organogenesis, nor in the foetal period.^18^

An increased copy number variation in azoospermia factor region c (AZFc) of the Y chromosome is a marker of spermatogenic failure, previously associated with radiation exposure. Andreassi *et al*. compared 193 male catheterization lab workers who received a dose of 1–10 mSv/year to 164 age-matched unexposed controls. They reported more instability in the Y chromosome AZFc region (1.53 ± 0.85 vs.1.02 ± 0.41; *P*-value = 0.0005). Although birth weight is considered a maternal factor, they reported that exposed male workers had a higher prevalence of low birth weight in their offspring (13 vs. 5.3%, *P*-value = 0.02; OR = 2.7; 95%CI: 1.1–6.3).^19^

Prior studies have demonstrated that gonadal dose to operators during FGI was well below known thresholds for gonadal injury, even after prolonged exposures.^20,21^ With the reduction of radiation dose using modern X ray devices and the use of scatter barrier protection, exposure levels today are much below the known threshold doses for infertility.^22^

### Risk of miscarriage

According to previous studies, doses below 500 mGy have not been shown to increase the risk of interruption of embryo implantation or foetal demise.^2,23^ Several studies have evaluated under-lead doses encountered by interventional cardiologists and the mean effective dose was 1.02 ± 0.73 mSv/year.^24–26^ Moreover, several studies specifically examined the dose encountered for pregnant interventional operators using the under-lead waist and thyroid dosimeter, and showed an average range of 0–0.1 mSv per month.^27–30^ This dose is much lower, than the dose needed to induce foetal loss.

Small retrospective studies have shown no difference in foetal loss rates between occupationally exposed women and the general population.^31^ However, there are no prospective studies on miscarriage among healthcare workers exposed to ionizing radiation.

Flight attendants, are a group of occupationally exposed women in a physically demanding profession who are exposed to a comparable level of radiation as in FGI (0.2–0.5 mSv per year, depending on travel routes). When adjusted for age, flight attendants did not experience an increased incidence of foetal loss compared with the general population.^32,33^

### Foetal risk

#### Deterministic vs. stochastic effects

Foetal radiation exposure can cause two types of adverse effects: deterministic (non-probabilistic) and stochastic (probabilistic). Deterministic effects occur after a threshold dose and include intrauterine growth restriction, miscarriage, mental retardation, low intelligence quotient (IQ), and congenital malformations. There is no threshold dose for stochastic effects, although the probability of their occurrence rises as the dose exposure increases. The most significant is childhood cancer.^17^ In the following sections we review the literature regarding risk of birth defects, childhood cancer, and long-term neurodevelopmental outcomes (*Table 1*).

**Table 1:** Effects of gestational age and radiation dose on radiation-induced teratogenesis^34,35^

	Effects	Estimated threshold dose^34^
*Gestational period*		
Before implantation (0–2 weeks after fertilization)	Death of embryo or no consequence	50–100 mGy
Organogenesis (2–8 weeks after fertilization)	Congenital anomalies (skeleton, eyes, and genitals)	200 mGy
	Growth restriction	200–250 mGy
*Foetal period*		
8–15 weeks	Severe intellectual disability (high risk)	60–310 mGy
	Intellectual deficit	25 IQ point loss/1000 mGy
	Microcephaly	200 mGy
16–25 weeks	Severe intellectual disability (low risk)	250–280 mGy

*Note*. Adapted from Guidelines for Diagnostic Imaging During Pregnancy and Lactation. American College of Obstetrics & Gynecology, 2017, p. 210–216.^34^

#### Risk of birth defects

Birth defects, malformations, and intellectual impairment are deterministic (dose-related) effects of radiation exposure that occur after exposure to high doses of radiation; the effects do not occur below known thresholds. Evidence for these dose thresholds is based mainly on studies on large-scale radiation disasters, animal studies, and outcomes in women exposed to radiation for medical treatment.^36,37^

The disabilities varied depending on the dose as well as gestational age, with the first trimester being the period of greatest foetal vulnerability to radiation.^38,39^ Exposure during Days 18–20 could result in ovum death. Doses between 1 and 2 Gy during organogenesis could cause severe foetal anomalies of the eyes and, nervous and skeletal systems. Doses >2 Gy could cause death. Exposure >50 days after conception can result in intrauterine growth retardation (*Table 2*).^34,37^ These doses are more than 10 times higher than the safety limits recommended by the American College of Obstetricians and Gynaecologists and NCRP.^34,37–39^ A large retrospective study looking at potential maternal occupational exposure to ionizing radiation during pregnancy found no association with birth defects overall.^35^

**Table 2:** Specific strategies for radiation protection during pregnancy in the catheterization laboratory

Category	Strategy
1. Time	Reduce time of procedures by avoiding unnecessary cines.
	Minimize fluoroscopy time. Reduce unnecessary catheters manipulations under fluoroscopy.
	Plan and study FGI in advance for cases with previous interventions or previous cines.
2. Source	In EP apply all technologies that allow to perform procedures without radiation.
	Use electro-anatomical mapping systems in EP whenever possible.
	Obtain appropriate training.
	Use lowest possible frame rate.
	Use low magnification.
	Avoid cine acquisition by using the ‘fluoro-save’ option.
	Use of collimation.
	Use short intermittent radiation instead of continuous.
3. Distance	Place the image detector as close as possible to the patient, to reduce scatter radiation.
	Use available patient dose reduction technologies.
	Wear your dosimeters and know your own dose.
	Avoid hands in the primary beam.
	Avoid being close to the X-ray tube.
	Minimize the use of extreme angulated projections.
	Minimize the use of projections with higher effective doses to the operator such as left anterior oblique-cranial.
4. Shielding	Always keep the X-ray tube under the table.
	*Protection devices*: lead glass goggles, thyroid protection, two-piece lead apron (jacket and skirt) to distribute weight and 0.5 mm equivalent width (overlapping two layers of lead) at the front and 0.25 mm on the back (>90% protection).
	*Ancillary shields*: Ceiling-mounted screen, table curtain, lead skirt over the patient’s waist and mobile floor shield, especially during cine acquisition.
5. Specific measures for fertility women	*Radiation cabin*: Zero-gravity, Biotronik, Germany and Cathpax Air, LemerPax, France.
	Double thickness of abdominal shielding.
	Improve work practices in the catheterization laboratory before pregnancy.
	Continue with the same practices as before the pregnancy but with greater care.
	Position yourself in a low-scatter area.

#### Risk of developing cancer

There are no well controlled studies on the effects of radiation on pregnant women and their children. The earliest data comes from the Oxford Survey of Childhood Cancer,^40^ which analysed all children with malignant diseases diagnosed in the UK from 1953 to 1981. During that period of time, pelvic radiographs were common practice to determine the best method of delivery. There was a significant risk for childhood leukaemia as well as an increased risk of central nervous system and solid tumours. However, more recent studies were not consistent with these findings.^41^ A meta-analysis published in 2008, examining prenatal exposure to diagnostic radiographs concluded statistically significant increase in risk of childhood leukaemias, but a causal interpretation has been questioned.^42^

In a large-scale study of radiologic technologists between 1921 and 1984, there was no evidence of an increased risk of childhood cancer among their children.^43^ A UK study of children born to radiology technologists showed similar rates of major congenital malformations, chromosomal abnormalities, and cancer as in the general population.^31^

In the absence of occupational exposure to radiation, the likelihood of having a baby without malformations and not developing childhood cancer is 95.93%. The probability of live birth without malformation or childhood cancer at 1 mGy conceptus dose above the average annual global natural background radiation of 2.4 mSv is 95.99% and at 5 mGy 95.88%. Thus these probabilities are almost identical.

#### Long-term effects

Long-term effects of prenatal low dose radiation on neurodevelopmental outcomes remain controversial. Previously published studies have shown an association between low dose prenatal radiation exposure and some cognitive functioning; mainly schizophrenia and verbal IQ.^44,45^ However, consequent and more recent studies were not consistent with these findings.^46,47^ Two comprehensive studies in Sweden and Norway collected exposure data following the Chernobyl accident in April 1986 combined with population-based registries to assess long-term effects of foetal exposure on neurodevelopmental outcomes. They found no evidence that radioactive fallout from Chernobyl in Norway, was associated with serious neurodevelopmental conditions (cerebral palsy, mental retardation, schizophrenia, epilepsy, or severe hearing and vision problems). Scant evidence was found for an association of higher radiation exposure (>0.024 mSv) during pregnancy with low school grades in mathematics at the age of 16.^48,49^

## Radiation exposure in the catheterization laboratory

### Basic concepts

The patient receives radiation exposure from the radiation tube, while the operator is exposed from scatter radiation mainly from the patient.^50^ Scattered radiation is about 10–20% of the patient ED,^51^ thus every effort should be made to decease the ED and all the scatter radiation.

Of all the personnel in the catheterization laboratory, the primary operator is the one who is most exposed and subject to the highest radiation dose due to the proximity to the radiation source and scatter from the patient. The radiation dose received by other working personnel is estimated to be 20 times lower than that of the first operator.^52^ In EP, many procedures are now being performed with less fluoroscopy, hopefully minimizing the issue of radiation exposure.^53,54^

**Figure 1:**
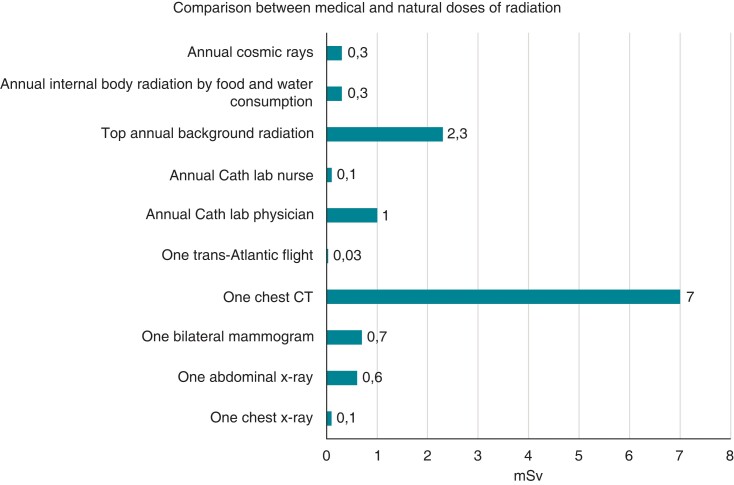
Comparison between medical and natural doses of radiation.

The ED for patients and operators during diagnostic coronary angiography is between 2–16 mSv (mean 7 mSv) and 0.02–38 µSv (mean 4.4 µSv), respectively; and higher during percutaneous coronary intervention 7–57 mSv (mean 15 mSv) and 0.17–31 µSv (mean 4.9 µSv), respectively (*Figure 1*). The trans-radial approach and less experience operators, usually are associated with higher amounts of radiation.^53^ The ED during ablations (atrial fibrillation, atrioventricular node, and ventricular tachycardia) is between 1.6–59.6 mSv (mean 15.2 mSv) for patients and 0.24–9.6 µSv (mean 2.7 µSv), for operators; and during pacemaker and implantable cardioverter-defibrillator devices is 1.4–17 mSv (mean 4 mSv), for patients, and 0.29–17.4 µSv (mean 4.8 µSv) for operators. Concluding that the dose received by operators is 100–2000 times lower than that received by the patient.^55,56^

Moreover, radiation doses in the cardiac laboratory have significantly decreased over time. One study showed a tendency to annual decrease of recorded occupational doses in the cardiac laboratory from a maximum of 16 mSv (mean 8.5 mSv) in 2007 to 3 mSv (mean 1 mSv) in 2017, for physicians. The nurse group showed a reduction of annual radiation from 1 mSv in 2011 to 0.1 mSv in 2017. As shown in *Figure 1*, a chest computer tomography exposure to a patient is around seven times higher than one year exposure of a cardiac lab physician.^54,55,57^

There are advances in x-ray machines, electro-anatomical mapping systems in EP, education and awareness campaigns that have resulted in a significant reduction in radiation over the recent years and allowed even for zero-fluoroscopy EP procedures.

### Shielding to reduce scatter radiation

Using a ceiling-mounted upper body shield in the correct position protects over 80% of scattered radiation and the lower body shield mounted on the side of the patient table rail provides more than 90% protection for the lower body; together, these ancillary shields provide more than 80% protection. But when the shields are not placed correctly (correct position: close to the patient’s surface and close to the puncture site) protection can be reduced to <50%.^51^ Additionally, a lead pelvic apron over the patient’s hips can reduce scattered radiation by an additional 23–42% for the operator^58–60^ in both the radial and femoral approach.

The European authorities recommend not to exceed around 1 mSv of exposure during pregnancy, on the contrary, the United States (US) authorities recommend not to exceed around 5 mSv during pregnancy and <0.5 mSv per month.^61^ These limits increase the baseline lifetime risk of developing cancer from 42.00% in the general population to 42.02% at the European limit and 42.20% at the US limit.^62^

To increase the abdominal shielding from the conventional recommendation of 0.5 mm to 1.0 mm thickness at the front, the options are to use 2 wrap-around lead skirts and 2 wrap-around lead jackets of 0.25 mm each with a total overlap of 4 layers on the front and 2 layers on the back, or use 2 conventional 0.5 mm aprons without overlapping. Double lead shielding increases protection from >90% to >95%,^63^ however apron weight must be taken into consideration in each specific case, due to the possibility of fatigue and exacerbation of musculoskeletal conditions.

Furthermore, foetal dose has been demonstrated by mathematical modelling and phantom testing to be <50% of the dose measured at the abdomen under the lead apron.^30^

### Dosimeters during pregnancy

It is recommended that two personal dosimeters be used in the catheterization laboratory, one on the trunk of the body (normally at chest level) inside the lead apron and one outside the apron, usually at neck (collar) or left shoulder level, but at least one dosimeter (the outside one) should be used.^64^ During pregnancy, the most valuable information can be obtained from the record of the dosimeter carried inside, and it is recommended to place a third dosimeter inside the lead apron at the abdomen level.^61^

The history of dosimetry data in the catheterization laboratory personnel, especially the record in women of childbearing age, must be known and the necessary corrections should be made in order to reduce exposure to the lowest recommended level before pregnancy. It is a powerful tool to improve the practices of the workers before they get pregnant, so that during pregnancy they can continue with the same line of work and the increase in the lead of the apron is only for backup and safety. The dosimetry results must be strictly notified to the worker to act in advance.

The foetus is most sensitive to radiation effects between 8 and 15 weeks of pregnancy (*Table 1*). This period is often before the pregnant worker announces her pregnancy to supervisors or co-workers, and therefore she may wish to request a foetal badge before actually declaring pregnancy. A worker who is considering pregnancy may also request an abdominal/waist badge. Reading from this badge can help to establish the likely conceptus dose that would be received with a normal work schedule.

In a retrospective study of 758 neurointerventional cases (47 cases/month before pregnancy and 46 cases/month during pregnancy, p = 0.862) of a fellow physician wearing an additional dosimeter under the apron in the middle of the lower abdomen and two lead apron skirts showed zero foetal radiation exposure without significant changes in the fellow’s case volume and fluoroscopy time.^28^

A case series of 5 operators from Spain doing FGI procedures throughout pregnancy found that the abdominal dose was well below the reference limit and in some cases no higher than the background radiation level.^65^ All pregnancies were uneventful. Similarly, a study at the Mayo Clinic found that the dose measured at the abdomen was below the detection limit in 82% of women; the only women to have measurable radiation detected were working in nuclear medicine, general X-ray or anaesthetics, but not in cardiology.^8^

## Radiation protection strategies during pregnancy

**Figure 2:**
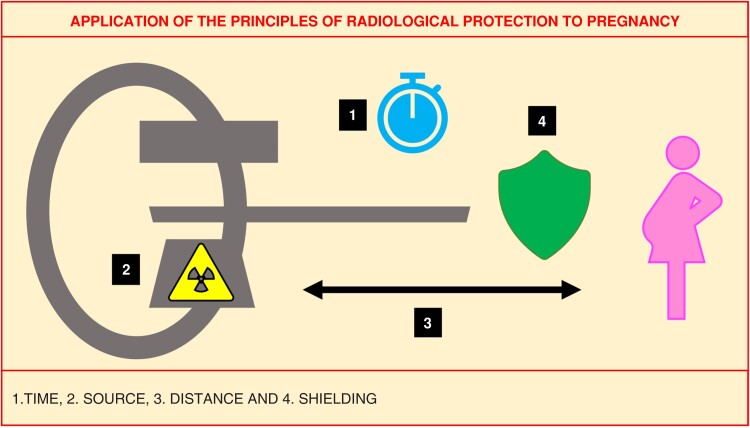
Application of the principles of radiological protection to pregnancy.

Understanding the radiation protection principles of time, distance and shielding is critical to minimizing exposure. All measures should be used to decrease radiation exposure measures (*Figure 2*). The pregnant women may require some more specific measures (*Table 2*).^63,66^  *Table 3* describes areas for future research.

**Table 3:** Areas for future research

What is the range of radiation exposure prior to pregnancy and especially during pregnancy with specific attention to the waist dosimeter. There is no long-term follow-up study providing data neither on exposed health professionals nor on their children. Such kind of cohort would be highly relevant. Prospective follow-up regarding long-term potential influence of radiation on fertility, rate of spontaneous abortions, small birth weight, birth defects, and learning difficulties and childhood cancer in the offspring's. What is the influence of real time dosimeters with audible alarm above a pre-set dose range on radiation exposure. Such devices may avoid incidental overexposure. What is the dose in men and its influence on conception, fertility, and child health. This is especially true in the pre-conceptional 3-month period.

There are additional concerns. The training period for interventional cardiology is longer than for other specialties. During this period it is not ‘career-convenient’ to undertake a pregnancy, which is instead postponed to a later period. Of course the older age to face a pregnancy represents, in itself, a risk for infertility problems and chromosomal pathologies. Furthermore, working in interventional cardiology during pregnancy does not only subject the woman to radiological risk but also to biological risk and also in this regard the laws are not uniform in the different countries.

Working with two lead coats during pregnancy increases the weight on the spine and lower limbs which, in itself, has already increased during gestation and influences on other effects on the health of the pregnant woman, for example venous insufficiency, thromboembolic risk, etc. Lead aprons are generally pre-formed on a male physique. During pregnancy and breastfeeding, the mammary glands are particularly sensitive to the effects of ionizing radiation and often the lead coats, perhaps worn in a larger size, leave a lot of uncovered space in the axillary area, and therefore the mammary area.

## Conclusions

A significant portion of the staff working in the catheterization laboratory are women. However, there is only a modest grow in the number of female physicians in the field of EP, interventional cardiology, and radiology. There are still concerned about the risk of radiation exposure during pregnancy. Although the evidence on infertility, miscarriage, foetal risk, and birth defects is limited, the available data point towards a very low risk of adverse events. Lack of accurate knowledge regarding the risk of occupational radiation exposure before conception and during pregnancy can cause fear leading to anxiety and altered career and family choices. Female cardiologists made more changes in their training and careers to reduce or avoid radiation exposure because of concerns related to risk to a developing foetus.

The biggest barrier to pregnant women in the catheterization/EP lab is ignorance and we tried to address some of important gaps in knowledge. Education around this issue is crucial for female cardiologists, both in terms of making an informed decision about staying in the catheterization laboratory during pregnancy and knowing how to monitor and reduce their radiation exposure, should they choose to do so. Education of catheterization laboratory directors, be them male or female, is also necessary. Finally, education of policy/law-makers is another important step to overcoming barriers to pregnant women in the catheterization laboratory.

Using all available strategies for radiation protection the risk for childbearing and pregnancy appears to be low and should not be the reason for women to avoid working and performing FGI. Understanding better the data, and using appropriate protection, can increase the number of women in the field of interventional radiology, EP, and interventional cardiology.

## Supplementary Material

euac158_Supplementary_Data

## Data Availability

The data underlying this article will be shared on reasonable request to the corresponding author.
