# Supplementary material for: Risk of ionizing radiation in pregnancy: just a myth or a real concern?
Source: Europace. 2022 Sep 20;25(2):270–6. doi: 10.1093/europace/euac158 (PMC10103573; doi:10.1093/europace/euac158)
Supplement: euac158_Supplementary_Data [file euac158_supplementary_data.docx]

**Supplementary Table 1. Commonly Used Measures of Radiation Exposure** (9)

| **Quantity** | **Units** | **Definition** | **Use** |
| --- | --- | --- | --- |
| Absorbed dose | Gy | The amount of energy locally deposited in tissue | Assesses the potential risk to a specific tissue |
| Effective dose | Sv | Attributed whole-body dose that produces the same whole-person stochastic risk as an absorbed dose to a limited portion of the body | Comparison of risks when doses are delivered to different organs in individuals |
| Air kerma | Gy | Sum of initial kinetic energies of all charged particles liberated by the *X* rays per mass of air | Assesses the level of hazard at the specified location |
| Exposure | mC×kg^—1^ | Total charge of ions of one sign produced by the radiation per unit mass of air | Assesses the level of hazard at the specified location |
| Equivalent dose | Sv | Quantity factoring in relative biological damage caused by different types of radiation | Most common unit used to measure radiation risk to specific tissues |

# Note. Adapted from ACC expert consensus document. Radiation safety in the practice of cardiology by Limacher MC, et al. 1998, p. 895 [9].
